# An examination of the mediating role of salt knowledge and beliefs on the relationship between socio-demographic factors and discretionary salt use: a cross-sectional study

**DOI:** 10.1186/1479-5868-10-25

**Published:** 2013-02-19

**Authors:** Rani Sarmugam, Anthony Worsley, Wei Wang

**Affiliations:** 1 School of Exercise and Nutrition Sciences, Deakin University, 221 Burwood Highway, Burwood, VIC, 3125, Australia

**Keywords:** Knowledge, Beliefs, Socio-demographic, Salt, Sodium, Mediation

## Abstract

**Background:**

Discretionary salt use varies according to socio-demographic factors. However, it is unknown whether salt knowledge and beliefs mediate this relationship. This study examined the direct and indirect effect of socio-demographic factors on salt knowledge and discretionary salt use in a sample of 530 Australian adults.

**Methods:**

An internet based cross-sectional survey was used to collect data for this study. Participants completed an online questionnaire which assessed their salt knowledge, beliefs and salt use behaviour. Mplus was used to conduct structural equation modelling to estimate direct and indirect effects.

**Results:**

The mean age of the participants was 49.2 years, and about a third had tertiary education. Discretionary salt use was inversely related to age (r=-0.11; p<0.05), and declarative salt knowledge (knowledge of factual information) scores (r = -0.17; p<0.01), but was positively correlated with misconceptions about salt (r = 0.09; p<0.05) and beliefs about the taste of salt (r = 0.51; p<0.001). Structural equation modelling showed age, education and gender were indirectly associated with the use of discretionary salt through three mediating pathways; declarative salt knowledge, misconceptions about salt and salt taste beliefs.

**Conclusions:**

Inequalities observed between socio-demographic groups in their use of discretionary salt use can potentially be reduced through targeted salt knowledge and awareness campaigns.

## Introduction

Higher intakes of dietary salt intake have been shown to increase blood pressure [1,2] and may have possible role in increased risk of stroke [3,4]. Elevated blood pressure plays a major role in the aetiology of cardiovascular disease [5].

A number of Australian studies have found that salt intake ranges between 6.4 g to 10 g per day [6-8], exceeding the recommended maximum amount of 6 g/day [9]. Similarly, data from U.S. and U.K. shows that the population salt intake exceeds the recommended amount [10,11].

Similar to other western diets [12-14], the food categories that contribute most to Australians’ salt consumption are processed foods including bread and cereal products (32%) followed by meat products and dishes (21%) [15]. It is estimated that processed foods account for about 80% of salt in the Australian diet while discretionary salt contributes about 20% [16]. In order to achieve its population intake target of 6 g salt per day, the UK Food Standards Agency proposed a salt reduction strategy which comprises both reduction of salt in major salt contributing food categories (such as bread and cereals) as well as discretionary salt intake (i.e. salt added to the food) [17,18].

In Australia, ischaemic heart disease and cardiovascular disease were the two leading causes of death in 2010 [19]. The prevalence of cardiovascular disease and coronary heart disease was higher among the Australians in the lowest socio-economic groups compared to those in the highest socio-economic groups [20,21]. Higher proportions of individuals with lower levels of education were diagnosed with hypertension compared to those with higher levels of education [20].

Differences observed in dietary behaviours and diet quality are often attributed to socio-demographic factors such as age [22], gender [23], education [23-25] and income [24,25]. Similarly, use of discretionary salt is also associated with socio-economic factors [26]. Individuals from lower income households, those with lower levels of education [26], males [27] and younger adults [28] have higher levels of discretionary salt use.

Apart from financial cost [29] and environmental factors such as access to healthier diets [30], social cognitive factors such as knowledge [31], self-efficacy [32], attitudes and beliefs [33] are often among the reasons attributed for the variation in dietary quality among the different socio-economic groups. For example, individuals with higher levels of education [31,34-37] and higher incomes [34,38] have been shown to have higher levels of nutrition knowledge. Similarly, women [31,39] and older people [35,38] tend to demonstrate higher levels of nutrition knowledge. More specifically, knowledge about salt has been found to be higher among older people and those with higher levels of education [40].

Although socio-demographic factors such as age, gender, education and income are indicators of health inequalities, unless there is a major societal change [26], little can be done in the short term to address or change these factors. Therefore, identification of modifiable, mediating factors may provide more feasible opportunities for interventions to reduce the disparities between the various socio-demographic groups.

To our knowledge, no study has examined the role of salt knowledge and beliefs as possible mediators between socio-demographic factors and discretionary salt use.

Given the importance of consumer knowledge as a likely influence on salt consumption and its importance for salt reduction policy monitoring [41], there is a need to clarify the role of salt knowledge and beliefs in relation to salt usage behaviour within the population.

Therefore, the aims of this study were 1) to examine the relationships between socio-demographic factors, salt knowledge, salt taste beliefs and discretionary salt use; and, 2) to determine the possible mediating roles of salt knowledge and salt taste beliefs in the relationships between socio-demographic status and discretionary salt use in an Australian adult population.

## Methods

Five hundred and sixty eight invitation emails were sent to a convenience sample of online members of a market research company’s research panel. The panel members were individuals who had registered and agreed to participate in surveys in return for reward points. The invitation email included the link to the survey website which contained the questionnaire which was completed online.

### Sample and procedure

The study population consisted of Australian adults above 18 years of age. The participants were invited to answer a self-administered online questionnaire, which could be completed at their convenience within 20 to 25 minutes. The study was approved by the University of Wollongong Research Ethics Committee (Ethics reference no: HE11/351).

The survey was kept open for seven days. During this period, a total of 574 individuals completed the survey; of these, 44 respondents were excluded for not meeting the screening criteria set to ensure data quality [42]. For example, respondents who completed the survey in less than one third of the average completion time were considered likely to have sped through the survey without giving much thought. Therefore, these responses were excluded from data analysis. A total of 530 usable questionnaires were used in the final analysis.

### Survey questionnaire

The salt knowledge and beliefs items formed part of a larger questionnaire, which examined salt knowledge and food purchasing behaviour.

Salt knowledge and beliefs about salt taste were measured using a validated salt knowledge questionnaire [43] which contained 25 items (see Additional file 1) relating to declarative knowledge which is defined as “awareness and understanding of factual information” [44] or also known as “know that” [44] knowledge (for example, knowledge about the properties of nutrients such as salt and risk factors associated with high salt intake) and procedural knowledge or “know how” knowledge [45]. In addition, it also assessed misconceptions about salt and health. The declarative knowledge section included questions about dietary recommendations, diet-disease relationships, and the salt content of commonly eaten foods while the procedural knowledge included questions on label reading [43].

### Salt knowledge scoring

All correct (i.e. accurate) responses were scored as one, while incorrect responses which included “don’t know” or “not sure” and non-responses were assigned a score of zero. Scores from three sections: dietary recommendations, diet-disease relationships, and salt content of commonly eaten foods were summed to form declarative knowledge scores and the sum of scores obtained from the label reading questions formed procedural knowledge scores. These scores were used for the subsequent analyses.

### Misconceptions about salt

Misconceptions about salt were assessed using three items (Additional file 1) with five-point Likert scales ranging from “certainly wrong” to “certainly true”. Responses which indicated misconceptions were scored as one, while other responses were assigned a score of zero. For example, a score of one was given if the respondent answered “certainly true” or “probably true” for the following item “Sea salt is better than table salt”. The scores were summed to derive a total score for misconceptions about salt. Higher scores indicate higher levels of misconceptions or “false beliefs” about salt.

### Beliefs about the importance of the taste of salt

Beliefs related to the taste of salt were assessed using two items; 1) “In general, low salt food tastes bad”, 2) “Salt should be used in cooking to enhance the taste of the food”. These belief items were measured on five-point Likert scales ranging from “certainly wrong” to “certainly true”. Reliability analysis showed that the two items formed one factor (Cronbach’s alpha = 0.56). The items were summed to derive a total beliefs score about the importance of the taste of salt. Higher scores indicate stronger beliefs about the importance of the taste of salt.

### Socio-demographic questions

Socio-demographic questions elicited information about gender, age, highest level of education and household income (Table 1).

**Table 1:** Socio-demographic characteristics of the study sample

		**Sample% (N)**	**Census**^a^**(%)**
**Gender**	Male	41.7 (221)	48.6^b^
	Female	58.3 (309)	51.4
**Age (years)**	18-20	2.8 (15)	3.6 ^b^
	21-30	15.5 (82)	17.6
	31-40	15.8 (84)	19.5
	41-50	18.1 (96)	19.6
	51-60	16.0 (85)	16.8
	61 - 70	23.2 (123)	11.1
	>70	8.5 (45)	11.8
**Highest level of education**	Left school at 16 years	25.7 (136)	
	Left school at 18 years	15.3 (81)	
	Technical and Further Education (TAFE) or college diploma, certificate or formal trade qualification	32.1 (170)	45.4^c,d,e^
	Bachelor degree/ Graduate Diploma / Graduate Certificate	19.6 (104)	24.5
	Postgraduate degree	7.4 (39)	4.9
**Employment**	Employed full-time	33.0 (175)	36.6^c,d,f^
	Employed part-time / casual	17.5 (93)	16.9
	Home duties / retired/ student	40.0 (212)	33.1
	Unemployed / looking for work	9.4 (50)	3.2
**Household income (AUSD)**	10,000 or less	4.9 (26)	
	10,001 to 20,000	11.9 (63)	
	20,001 to 40,000	18.1 (96)	
	40,001 to 60,000	17.7 (94)	
	60,001 to 80,000	15.3 (81)	
	80,000 to or 100,000	13.4 (71)	
	Over 100,001	18.7 (99)	

^a^ Based on 2006 Census data [46]; ^b^ based on census data for population aged 18 and above; ^c^ based on individuals aged 15 years and over who stated completed qualification; ^d^ Denotes slight variation of categories between survey and census; ^e^ total percentages do not add up to 100% due to individuals who did not state or inadequately described their level of education; ^f^ total percentages do not add up to 100% due to individuals who have not stated their employment status.

### Discretionary salt use

Discretionary salt use was measured by two questions which had been used in a previous Australian National Nutrition Survey [47,48]. Participants were asked to indicate the frequency of their salt use at the table and in cooking based on four response categories “always”, “usually”, “sometimes” and “never or rarely” (Table 2). Scores were assigned according to the frequencies (1 for never, 2 for sometimes etc.). Higher scores indicated higher frequency in engaging in particular behaviours. The scores were then summed to reflect discretionary salt use. Higher scores indicate higher frequency of salt use.

**Table 2:** Self-reported frequencies of discretionary salt use

	**Add salt in cooking (%)**	**Add salt at the table (%)**	**Add salt after cooking NHS 2001**^**a**^**(%)**
Never/Rarely	35.1	48.1	54.9
Sometimes	29.2	26.2	19.5
Usually	23.6	17.0	25.5
Always	11.1	8.7	-
Do not prepare own meals	0.9	-	

^a^ Based on National Health Survey (NHS) 2001 data [47].

### Data analysis

**Figure 1:**
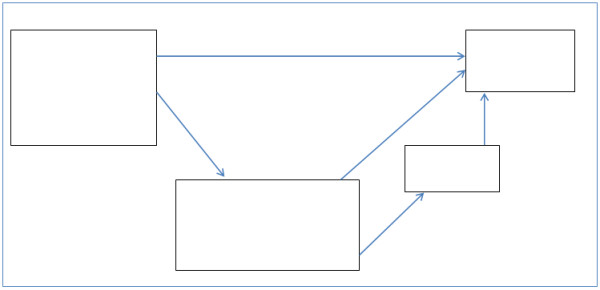
Theoretical model.

Descriptive statistics and Spearman bivariate correlations were calculated using SPSS Statistics version 18.0. Structural equation modelling was conducted using Mplus version 6.11 [49]. A path analysis was conducted based on the proposed theoretical model shown in Figure 1.

Mediation analyses were conducted based on the approach suggested by Hayes [50] which allows testing for indirect effects in the absence of direct associations between the independent and dependent variables. In addition, the magnitude of the of indirect effect was calculated as the ratio of indirect effect to the total effect and expressed as a percentage [51,52].

Model fit was tested using the Tucker-Lewis (TLI) and comparative fit (CFI) indices, standardised root mean square residual (SRMR) and root mean square error of approximation (RMSEA). Models were deemed to be acceptable when the fit indices met the following criteria; TLI and CFI >0.95, SRMR <0.08 and RMSEA <0.06 [53]. Because of their non-normal distributions, data were analysed using MLR (maximum likelihood parameter estimates with standard errors and a chi-square test statistic) that are robust to non-normality.

## Results

### Socio-demographic characteristics of the study participants

Table 1 shows the socio-demographic characteristics of the study participants. Slightly more than half of the respondents (58.3%) were female and about a third of the respondents (27.0%) had tertiary education. Almost half of the respondents’ had annual household incomes above $60,000.

### Discretionary salt use

Almost half (48.1%) of the respondents reported that they never or rarely added salt at the table, and only 35.1% of the respondents reported that they never or rarely added salt in cooking (Table 2). About a third of the study sample reported that they usually or always used salt in cooking (34.7%) and at the table (25.7%).

### Relationship between socio-demographic indicators with knowledge, beliefs and discretionary salt use

Table 3 shows the associations between socio-demographic indicators and salt knowledge, salt taste beliefs and discretionary salt use. Salt use was negatively correlated with age (r=-0.11; p<0.05), declarative salt knowledge scores (r=-0.17; p<0.001), and positively associated with the misconceptions score (r=0.09; p<0.05) and salt taste beliefs (r=0.51; p<0.001). However no significant association was found between procedural knowledge scores and salt use.

**Table 3:** Correlations between total salt knowledge scores and beliefs related to taste of salt scores with discretionary salt use

	**1**	**2**	**3**	**4**	**5**	**6**	**7**	**8**	**9**
1. Age									
2. Gender^┼^	-0.11^*^	1							
3. Education	-0.17^***^	-0.09^*^	1						
4. Income	-0.31^***^	-0.04	0.28^***^	1					
5. Salt use	-0.11^*^	-0.06	0.01	0.04	1				
6. Declarative knowledge	0.11^*^	0.09^*^	0.13^**^	0.06	-0.17^***^	1			
7. Procedural knowledge	-0.09^*^	0.04	0.12^**^	0.16^***^	-0.04	0.21^***^	1		
8. Misconceptions	0.00	-0.03	-0.14^**^	-0.08	0.09^*^	-0.24^***^	-0.24^***^	1	
9. Salt taste beliefs	-0.07	-0.13^**^	0.02	-0.02	0.51^***^	-0.12^**^	-0.11^*^	0.21^***^	1

^**┼**^Gender (1= male, 2= female); ***p<0.001,**p <0.01, *p<0.05.

### Structural equation modelling

**Figure 2:**
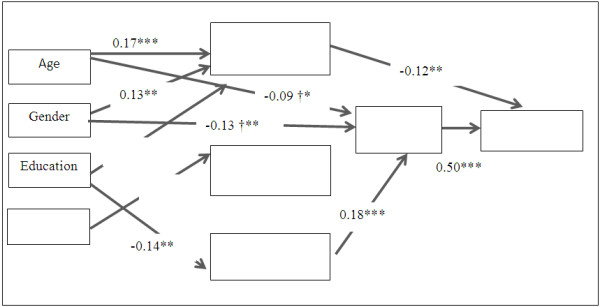
**Standardised regression co-efficient based on final model.** Gender is coded as 1= male, 2 = female; *p<0.05, **p<0.01, ***p<0.001; †path included in the analysis based on suggested modification indices; only statistically significant (p<0.05) paths are shown in this figure. Indirect paths are reported in Table 4 due to space limitations.

Structural equation modelling based on the proposed theoretical model (Figure 1) showed the data were a poor fit (χ^2^(4) = 13.07, p=0.01, CFI = 0.97, TFI = 0.78, SRMR = 0.02, RMSEA =0.07). The model was retested with additional paths between gender, age and salt taste beliefs based on the suggested modification indices and previous literature which indicated there are differences in food beliefs between genders [54] and age groups [55]. The final model (Figure 2) showed that the data were a good fit (χ^2^(2) = 1.20, p=0.05, CFI = 1.00, TFI = 1.03, SRMR = 0.01, RMSEA =0.00).

The model (Figure 2) explained 27.7% of variation in discretionary salt use. Age (β=0.17, p<0.001), gender (β=0.13, p<0.01) and education (β=0.15, p<0.01) were directly associated with declarative salt knowledge scores, with older adults, females and those with higher levels of education more likely to have higher levels of declarative salt knowledge while household income (β=0.13, p<0.01) was positively associated with procedural knowledge. Only education was found to have a significant inverse relationship with the misconceptions about salt (β=-0.14, p<0.01).

Of the socio-demographic predictors, only age and gender were directly associated with salt taste beliefs. Older people (β=-0.09, p <0.05) and females (β =-0.13, p<0.01) were more likely to have lower levels of salt taste beliefs. Salt taste beliefs had the strongest positive association (β = 0.50, p<0.001) with salt use followed by declarative knowledge (β =-0.12, p<0.01).

### Mediation analysis

Table 4 shows the direct and indirect relationships between socio-demographic factors, declarative salt knowledge, misconceptions and beliefs with discretionary salt use.

**Table 4:** Indirect associations of socioeconomic indicators and knowledge with salt us

	**Mediator 1**	**Mediator 2**	**Beta**	**SE**	**% mediation**^**a**^
Education	Declarative knowledge		-0.02	0.01	50
	Misconceptions	Salt taste beliefs	-0.01	0.01	25
Total indirect effect			-0.03	0.01	
Total effect ^ns^			-0.04	0.04	
Age	Declarative knowledge		-0.02	0.01	18
	Salt taste beliefs		-0.04	0.02	36
Total indirect effect			-0.07	0.02	
Total effect			-0.11	0.05	
Gender	Declarative knowledge		-0.02	0.01	
	Salt taste beliefs		-0.06	0.02	
Total indirect effect			-0.09	0.02	
Total effect ^ns^			-0.08	0.04	
Declarative knowledge^b^			-0.12	0.04	
Total indirect effect			-0.02	0.02	13
Total effect			-0.15	0.05	
Misconceptions	Salt taste beliefs		0.09	0.02	
Total indirect effect			0.09	0.02	
Total effect ^ns^			0.05	0.05	

Note: only statistically significant (p<0.05) paths are shown in this table.

ns: not significant; ^a^Ratio of indirect effect to total effect is only calculated when total effect is larger than indirect effect to avoid ambiguous estimates [52]; ^**b**^Indicates direct effect.

Education had a significant negative indirect effect on salt use (β =-0.03, p<0.05) through two paths 1) declarative knowledge which accounted for 50% of the total effect of education on salt (β = -0.02, p<0.05) and 2) misconception about salt and salt taste beliefs which accounted for 25% of the total effect (β =-0.01, p<0.05). Similarly, age had a significant negative indirect effect on salt use through two paths 1) declarative knowledge (β =-0.02, p<0.05) and 2) salt taste beliefs (β =-0.04, p<0.05). More than half (54%) of the total effect of age on salt use was mediated by these two paths. The effect of gender on salt use was mediated by salt taste beliefs (β =-0.06, p<0.01). No significant direct or indirect relationship was observed between household income and salt use. Declarative knowledge demonstrated a negative direct effect on salt use (β = -0.12, p<0.01). Misconceptions about salt had a significant indirect relationship via salt taste beliefs with salt use (β =0.09, p<0.001).

## Discussion

To our knowledge this is the first study which has examined the mediating effects of salt knowledge and salt taste beliefs on socio-demographic factors and discretionary salt use using a psychometrically validated salt knowledge questionnaire.

The proportion of respondents who reported always or usually adding salt at the table in this study (25.7%) was similar to the proportion of respondents who reported usually adding salt after cooking in National Health Survey 2001 (25.5%) [47]. The findings of this study demonstrated that age, gender and education influenced discretionary salt usage indirectly through salt knowledge (i.e. declarative knowledge), misconceptions and beliefs about the importance of the taste of salt. These findings are similar to other studies which have shown that nutrition knowledge mediates the relationships between socio-demographic factors and diet quality [56] and fruit and vegetables [57]. In addition, similar to previous findings [55] results of this study supported the importance of the role of beliefs about taste in use of discretionary salt.

However, contrary to previous findings [26], we did not observe any significant relationship between income and discretionary salt use. There is a possibility that the lack of direct relationship observed here might be caused by the interactions between the socio-demographic variables as shown in previous studies [58]. For example, income may mediate the relationship observed between education and salt use due to higher income being associated with higher levels of education.

Of the three components of knowledge, only declarative knowledge was directly and indirectly associated with salt use while misconceptions were associated with salt use indirectly through beliefs. Despite the postulated importance of procedural knowledge in dietary behaviour [39,45,59], and previous studies which showed that individuals who use food labels tend to exhibit healthier dietary practices [60,61], no relationship was observed in this study between procedural knowledge and discretionary salt use. Possibly the questions used to assess procedural knowledge were not being directly related to the use of discretionary salt (the questions in this study were related to the reading of food labels). Alternatively, this could also suggests that components of declarative knowledge i.e. understanding dietary recommendations, diet-disease relationships and knowing the salt content of commonly eaten foods are important determinants of discretionary salt use and should be included in salt education campaigns.

### Implications for nutrition policy and communication

Evidence of modifiable factors such as knowledge and salt taste beliefs which mediate the influence of socio-demographic factors on discretionary salt use provides an opportunity for the design of effective behavioural change interventions which operate on these mediators. For example, at present, most national salt reduction strategies include consumer education and awareness campaigns [62]. Thus, the identification of cognitive mediators in this study would facilitate the creation of tailored nutrition education programmes to suit the needs of smaller segments of population. Higher use of the internet is increasingly making it possible to deliver cost effective and more interactive tailored nutrition education programme [63].

Although the influence of taste beliefs in the current study was limited to the use of discretionary salt, consumers’ perceptions about poor tasting, low salt products has been noted as one of the reasons which led to poor uptake of salt reduced products [64], and to the decision to reintroduce salt into salt reduced products [65]. This suggests that there is a need to correct the misconceptions and beliefs which exist around the use of salt in food. Changes in consumer salt taste beliefs are likely to induce changes in salt usage behaviour which might lead to the alteration of taste preferences and eventually acceptance and preference for lower salt products [66,67].

Most countries with salt reduction initiatives have a voluntary programme for food manufacturers to reduce salt content in processed foods [62]. Even though this approach has been shown as a cost- effective way to reduce salt in the diet [68], it may take time and may vary between food companies within the same food category [69] and between countries [12,18,69]. Therefore, until the time when there is widespread availability of food products with lower amounts of salt, active participation from consumers will be required to reduce the salt in their diet. This includes reading food labels to choose products with lower salt content and reduce the amount of discretionary salt use.

Salt reduction initiatives should include both components; working with food industries to reformulate food products and educating consumers to reduce discretionary salt to ensure gradual reduction in preference for salt taste [28,70]. In addition, health educators may also need to consider the most appropriate approach towards education and awareness campaigns i.e. whether to focus on a single nutrient (dietary salt) or the overall diet and behavioural factors associated with hypertension [71].

### Limitations and future research directions

This study has several limitations. First, as a cross-sectional study, the findings can only be used to examine associations and not to draw inferences regarding causality. Further, results of this study might not be generalisable to the whole population due to use of convenience sampling. However, the sample appears to be more representative of the general population than samples from other surveys in which [37,39,72] female respondents or those with tertiary education are often over-represented. In contrast, in the present sample, the proportions of female respondents and those with tertiary education were similar to those in the general population [46].

Second, we have used self-reported use of table salt and salt in cooking as measures of discretionary salt use. Even though self-reported use of salt has been found to be correlated with actual behaviour [73], a more objective measure such as that provided by the lithium-marker technique [74] should be considered for future studies.

Third, discretionary salt use contributes less than 20% of the total amount of dietary salt in average person’s diet [16,75] while processed foods account for about 80% of the salt in Australian diet. Therefore, measurement of discretionary salt represents only a small amount of consumers’ dietary salt intake. Further, it is possible that use of discretionary salt may be lower when foods with high salt content are consumed or prepared and therefore it may potentially underestimated total salt intake. However, previous studies have shown that that higher use of discretionary salt was associated with higher total salt intake [76,77]. For example, analysis of 24-hour urinary sodium excretion of Australian adults between the age of 50 to 75 years found that those who reported use of salt in cooking had higher urinary sodium excretion than those who did not [78].

Fourth, the model only explained about a third of the variation in discretionary salt use. Therefore, future studies should be extended to study other factors which may mediate the relationship between socio-demographic factors and use of discretionary salt such as self-efficacy [32] and attitudes [33] and salt taste preferences. In addition, interactions between socio-demographic predictors such as education, household income and differences between the genders should also be explored. There is also a possibility that the total variation explained by the model can be increased with the addition of processed food items as outcome variables, especially those which were used to assess salt knowledge levels (e.g. processed meat, cheese).

Fifth, it should be noted that the questions on procedural knowledge in this study only focused on label reading and not on discretionary salt use. Therefore, caution should be exercised when interpreting all results pertaining to procedural knowledge. Future studies should extend the scope of procedural knowledge to clarify whether the reason for a non-significant relationship observed between procedural knowledge and discretionary salt use was due to limited scope of procedural knowledge or declarative knowledge is indeed a more important component of knowledge in predicting salt use.

Finally, since only two items were used to measure the salt taste beliefs construct in this study, as expected, the internal reliability of the beliefs construct was moderately low [79]. Despite this low reliability, we found a moderate correlation between beliefs and discretionary salt use. Therefore, future studies should employ a greater number of belief items to further examine the relationship between beliefs and salt use. There is a possibility that a scale with higher reliability may increase the relationships observed between beliefs and salt use.

## Conclusion

In conclusion, this study has demonstrated that declarative salt knowledge, salt beliefs and misconceptions mediate the relationships between age, gender and education with discretionary salt use. The study findings provide health promoters with opportunities to design targeted education and awareness campaigns. However, the study needs to be replicated to confirm the applicability of the findings in other populations.

## Competing interest

The authors declare that they have no competing interest.

## Authors’ contributions

RS and AW were responsible for the design of study. RS analysed and wrote the paper with contributions from AW. WW assisted in the data analyses. All authors approved the final draft of the manuscript.

## Supplementary Material

Additional file 1 List of salt knowledge questions. Click here for file
