# Supplementary material for: An examination of the mediating role of salt knowledge and beliefs on the relationship between socio-demographic factors and discretionary salt use: a cross-sectional study
Source: Int J Behav Nutr Phys Act. 2013 Feb 19;10:25. doi: 10.1186/1479-5868-10-25 (PMC3610230; doi:10.1186/1479-5868-10-25)
Supplement: Additional file 1 — List of salt knowledge questions. [file 1479-5868-10-25-S1.doc]

List of salt knowledge questions

| **Category** | **Section/Question** |
| --- | --- |
| **Declarative knowledge** | **Dietary recommendations:** Relationship between salt and sodium; How many grams of salt is equivalent to one teaspoon of salt?; A product is considered as ‘low in salt’ when it contains..; Maximum recommended daily amount of salt for an adult in Australia. |
|  | **Conditions which might be associated with high salt intakes:** High blood pressure; High blood sugar; Stroke; Kidney disease; Osteoporosis. |
|  | **Salt content of commonly eaten foods:** White bread; Bacon; White rice (boiled); Beef steak (uncooked); Mix vegetables (frozen); Corn flakes; Vegemite; Cheddar cheese (Processed). |
|  | Salt is naturally present in fresh food; Fast foods are high in salt; Bread is one of the main sources of salt in Australians’ diets. |
| **Common misconceptions** | Sea salt is better than table salt; Drinking more water can neutralize salt in my diet; Cutting down on salt causes leg cramps. |
| **Procedural knowledge** | **Label reading:** Which pasta sauce has the highest salt content; If you see a TICK logo on a packet of breakfast cereal, what do you think about the product? |
| *readers who wish to obtain the list of questions may contact the first author of this study. | |
